# HERC 1 Ubiquitin Ligase Mutation Affects Neocortical, CA3 Hippocampal and Spinal Cord Projection Neurons: An Ultrastructural Study

**DOI:** 10.3389/fnana.2016.00042

**Published:** 2016-04-18

**Authors:** Rocío Ruiz, Eva María Pérez-Villegas, Sara Bachiller, José Luis Rosa, José Angel Armengol

**Affiliations:** ^1^Department of Biochemistry and Molecular Biology, University of SevilleSeville, Spain; ^2^Department of Physiology, Anatomy and Cell Biology, University Pablo de OlavideSeville, Spain; ^3^Departament de Ciències Fisiològiques II, IDIBELL, Campus Bellvitge, University of BarcelonaBarcelona, Spain

**Keywords:** autophagy, cerebellum, cerebral cortex, hippocampus, neuron, proteasome, spinal cord, ubiquitin

## Abstract

The spontaneous mutation *tambaleante* is caused by the Gly483Glu substitution in the highly conserved N terminal RCC1-like domain of the HERC1 protein, which leads to the increase of mutated protein levels responsible for cerebellar Purkinje cell death by autophagy. Until now, Purkinje cells have been the only central nervous neurons reported as being targeted by the mutation, and their degeneration elicits an ataxic syndrome in adult mutant mice. However, the ultrastructural analysis performed here demonstrates that signs of autophagy, such as autophagosomes, lysosomes, and altered mitochondria, are present in neocortical pyramidal, CA3 hippocampal pyramidal, and spinal cord motor neurons. The main difference is that the reduction in the number of neurons affected in the *tambaleante* mutation in the neocortex, the hippocampus, and the spinal cord is not so evident as the dramatic loss of cerebellar Purkinje cells. Interestingly, signs of autophagy are absent in both interneurons and neuroglia cells. Affected neurons have in common that they are projection neurons which receive strong and varied synaptic inputs, and possess the highest degree of neuronal activity. Therefore, because the integrity of the ubiquitin-proteasome system is essential for protein degradation and hence, for normal protein turnover, it could be hypothesized that the deleterious effects of the misrouting of these pathways would depend directly on the neuronal activity.

## Introduction

The mutation *tambaleante* (*tbl/tbl*) appeared spontaneously in the colony of the Pasteur Institute (Paris, France) at the end of the 1980s. Previous studies have correlated the motor phenotype of the mutation as characterized by tremor, unstable gait, and abnormal posture of the hind limbs, with an ataxic syndrome similar to others suffered by mutant mice that have lost cerebellar Purkinje cells (Wassef et al., 1987; Rossi et al., 1995). Purkinje cell loss takes place from the 2nd month of postnatal life on, the date from which ataxic signs become more and more evident throughout the animal’s lifespan, coinciding with the progressive death of the Purkinje cells, whose number decreases dramatically to almost disappearing in 1-year-old mice (Dusart et al., 2006). However, Purkinje cells seem not to be the exclusive target of the mutation; thus, we have recently reported that 1-month-old *tbl/tbl* mice—that is, before the beginning of Purkinje cell degeneration—present alterations of their motor performance. This motor impairment was closely related to three main alterations of the neuromuscular junction: (i) the reduction of the motor end-plate size; (ii) the decrease of neuromuscular activity efficiency *in vivo*; and (iii) the impairment of the evoked neurotransmitter release (Bachiller et al., 2015).

The *tbl/tbl* mutation was molecularly characterized as linked to the alteration of the HERC1 (HECT domain and RCC1 domain) E3 ubiquitin ligase protein, in which the Gly483Glu substitution induces the protein overexpression responsible for the *tbl/tbl* phenotype (Mashimo et al., 2009). HERC1 E3 ubiquitin ligase protein belongs to the ubiquitin–proteasome system (UPS; Hegde and Upadhya, 2007; van Tijn et al., 2008). UPS plays a key role in the protein degradation pathway essential for neuronal homeostasis, and whose alteration has been postulated as involved in the pathogenesis of several neurodegenerative disorders, such as Alzheimer’s, Huntington’s, and Parkinson’s diseases (de Vrij et al., 2004; Upadhya and Hegde, 2005; Rubinsztein, 2006; Hegde and Upadhya, 2007; van Tijn et al., 2012), or the spinal and bulbar muscular atrophy (SBMA) X-spinal muscular atrophy (Rusmini et al., 2007; Ramser et al., 2008; Deng et al., 2011; Dlamini et al., 2013).

A high number of cerebellar mutant mice, irrespective of the molecular origin of their mutations, also possessed alterations in other regions of their central nervous systems (for a review, see Porras-García et al., 2013). These data, together with the fact that neuromuscular transmission was also affected in the *tbl/tbl* mutation, led us to investigate the possibility that other regions of the *tbl/tbl* nervous system could also be damaged.

## Materials and Methods

### Animals

*Tambaleante* mice were obtained by breeding pairs of *tbl* carrier mice. Mice were genotyped by PCR as described previously (Mashimo et al., 2009), and 1- to 4-month-old *tbl/tbl* mice and isogenic wild type (*wt*) mice were used. Animal care was according to current Spanish legislation RD 53/2013 governing experimental animals (BOE 08/02/2013), and under the approval of the ethical committees of our universities.

### Electron Microscopy Procedure

Mice were deeply anesthetized with an overdose of pentobarbital (80 mg/kg i.p.) and perfused intracardially with 1% glutaraldehyde and 1% paraformaldehyde in phosphate buffer 0.1 M (PB, pH 7.4). After dissection, the brain and spinal cord were fixed overnight in the same fixative at 4°C. Coronal slices of brain, cerebellum, and spinal cord were cut and postfixed in 2% OsO_4_ in PB at room temperature. Subsequently, the tissue was stained with 2.5% uranyl acetate in 70% ethanol at 4°C, dehydrated with ethanol and acetone at room temperature, and embedded in Durcupan (Fluka^®^). Semithin sections 1.5 μm thick were stained with 1% pyronin G and 1% toluidine blue. Ultrathin sections 60–70 nm thick were obtained from selected areas in a Leica UC6 ultramicrotome and collected on 200 mesh copper grids. To avoid the possibility of artifacts or precipitates, ultrathin sections were observed without uranyl acetate and lead citrate counterstaining on a Zeiss Libra 120 transmission electron microscope. Plates of figures were made using the software Photoshop 7.0 (Adobe®) without any additional correction of the microphotographs.

### Immunohistochemical Procedure

Four-month-old *wt* and *tbl/tbl* mice were deeply anesthetized with an overdose of pentobarbital (80 mg/kg i.p.) and perfused intracardially with 4% paraformaldehyde in PB. After dissection, the brains were fixed overnight in the same fixative at 4°C, and immersed in 30% sucrose in PB at 4°C until they sank. Coronal 30 μm thick sections were cut with a freezing microtome and collected in PBS. The immunohistochemical procedure has been previously reported (Bachiller et al., 2015), with the difference that for permeabilization 0.1% saponin was used instead of 0.25% Triton X-100. The primary antibodies used for double labeling were: a rabbit polyclonal antibody against Calbindin D-28k (1:5000, Swant, Cb-38a), a mouse monoclonal antibody against NeuN (1:250, Millipore, mab377), a mouse monoclonal antibody against p62 (1:100, Santa Cruz Biotechnology, Inc., SQSTM1 (D-3): sc-28359), a mouse monoclonal antibody against beclin-1 (1:100, abcam, ab62557), and a rabbit polyclonal antibody against light chain 3 (LC3; 1:100, Cell Signaling Technology #2275). The secondary antibodies used were: Alexa Fluor^®^ 488 donkey-anti-rabbit (1:500, Invitrogen A21207) to detect calbindin, Alexa Fluor^®^ 488 donkey-anti-mouse (1:500, Invitrogen A21202) to detect NeuN, Alexa Fluor^®^ 594 donkey-anti-mouse (1:500, Invitrogen A21203) to detect p62 and beclin-1, and Alexa Fluor^®^ 594 donkey-anti-rabbit (1:500, Invitrogen A21207) to detect LC3. Images were acquired in an upright Leica DM 2500 confocal laser scanning microscope. Plates of figures were made using the software Photoshop 7.0 (Adobe^®^) without any additional correction of the microphotographs.

### Quantitative Analysis

The procedure followed to quantify beclin-1, LC3 and p62 immunoreactivity densities was previously reported (Bachiller et al., 2015). Briefly, during image acquisition, an alternating sequence of laser pulses was used to activate the different fluorescent probes. Images were taken using a 40× oil-immersion objective with a numerical aperture of 0.65. Images from *wt* and *tbl/tbl* hippocampus and cerebral cortex were obtained under similar conditions (laser intensities and photomultiplier voltages), and on the same session. Quantitative analysis of the fluorescent labeling density was performed offline with FijiImageJ (W. Rasband, National Institutes of Health). Size of measured areas was determined automatically by defining outline masks based on brightness thresholds from maximal projected confocal images. The Student’s *t* test (two tailed) was used to compare *tbl/tbl* and *wt* counts. A *p* < 0.05 value was considered as statistically significant.

## Results

**Figure 1:**
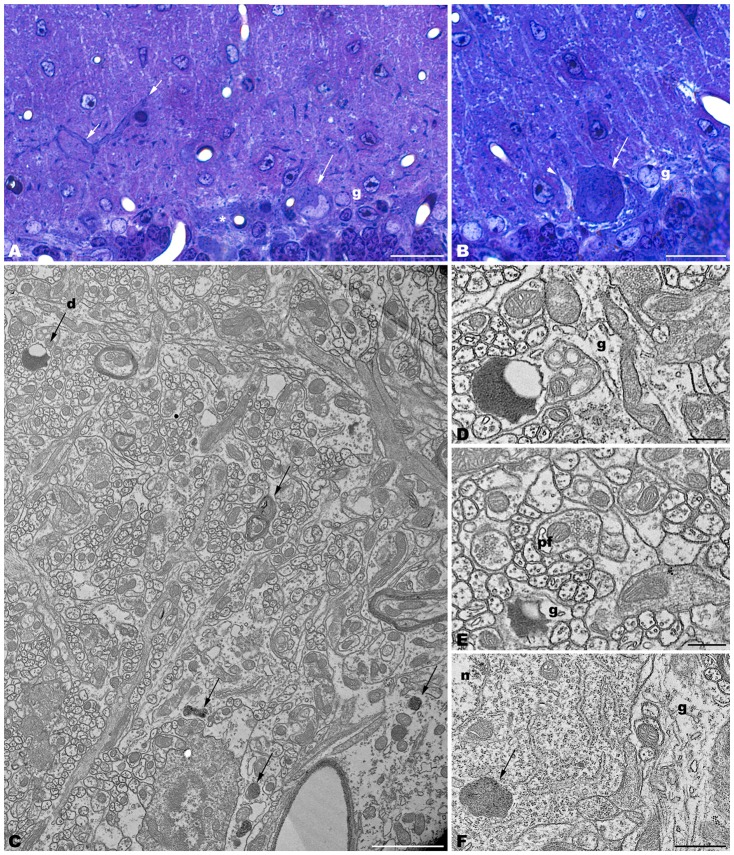
**Parasagittal sections through the cerebellar cortex of 4-month-old *tbl/tbl* mice.** 1.5 μm-thick sections illustrate that disappeared Purkinje cells are substituted by glial Golgi-epithelial cells (g, **A,B**). Remaining degenerating Purkinje cells possess degenerative dark accumulations within the cytoplasm of the soma (**A,B**, asterisk, arrows) and thick, dark dendritic trees (**A**, small arrow). Note the swelling of glial processes surrounding Purkinje cells (**B**, arrowhead). Degenerative signs consisting of lysosomes, electron-dense debris, and autophagosomes with different degrees of evolution (arrows in **C–F**), are present in the dendrites of the molecular layer and in the Purkinje cells’ cytoplasm (arrows, in **C,F**). Necrotic debris is also engulfed by glial cell processes (**D,E**, **g**). n, nucleus of a Purkinje cell. Pf, parallel fiber. Bars = 20 μm **(A,B)**, 2 μm **(C)**, and 0.5 μm **(D–F)**.

**Figure 2:**
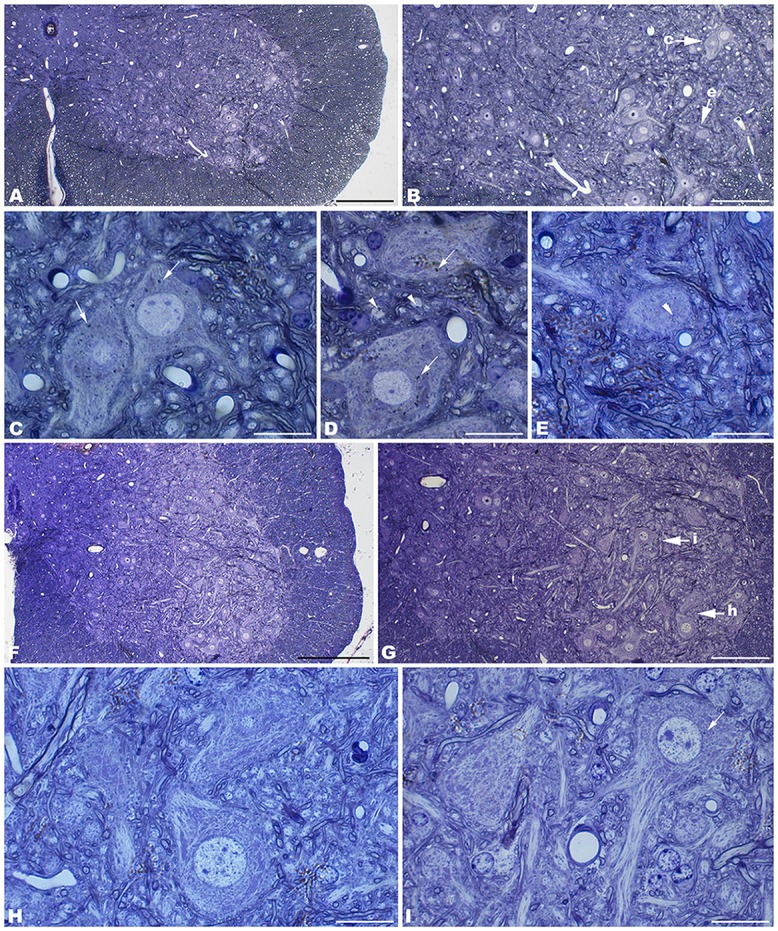
**Coronal sections through the spinal cord of 4- (A–E) and 1- (F–I) month-old *tbl/tbl* mice.** In the older mutant mice, abundant dark accumulations are observable within the cytoplasm of the cell soma (arrows in **C,D**) and the dendrites (arrowheads in **D,E**) of the motor neurons. In contrast, few of these dark aggregates can be found in the younger animals (arrow in **I**). Bars = 200 μm **(A,F)**, 100 μm **(B,G)**, and 20 μm (**C–E,H,I**).

**Figure 3:**
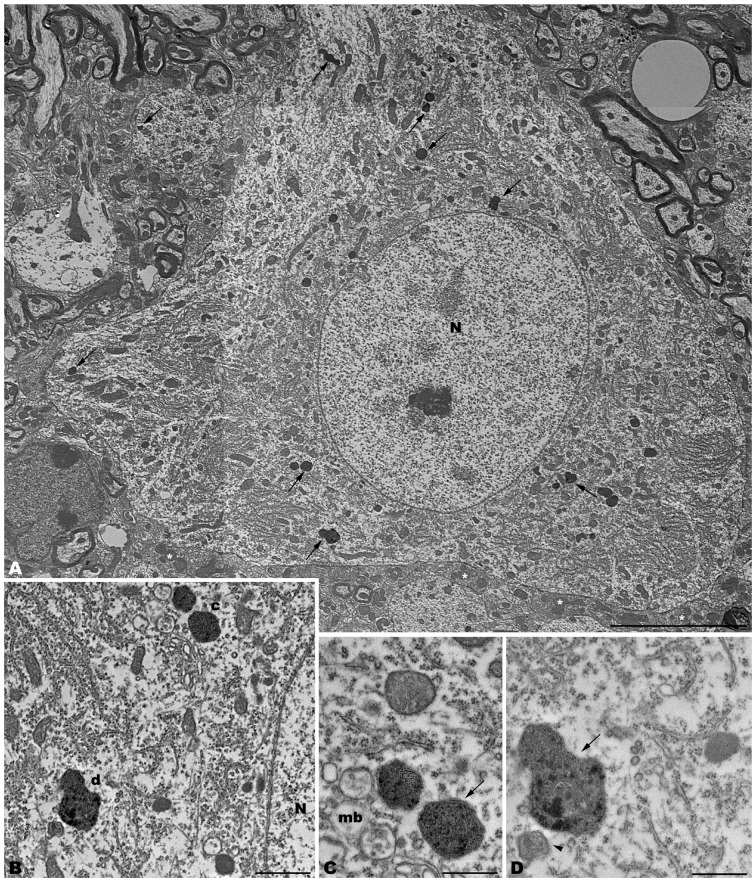
**Photomontage of a motor neuron from the spinal cord of a 4-month-old *tbl/tbl* mouse.** Arrows indicate lysosomes with different degrees of evolution distributed throughout the cell soma and dendritic cytoplasm **(A–D)**. Multivesicular bodies (mb), incipient autophagosomes (arrowhead), and empty vacuoles are often observed near the lysosomes **(B–D)**. Note that axosomatic and axodendritic synapses present an unaltered morphology (asterisks in **A**). Bars = 5 μm **(A)**, 1 μm **(B)**, and 0.5 μm **(C,D)**.

**Figure 4:**
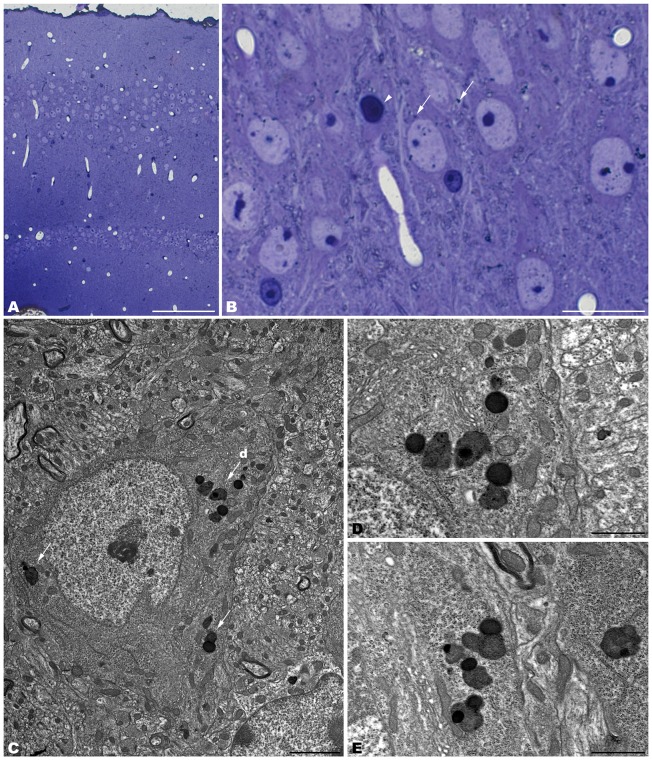
**Coronal sections through the CA3 of the hippocampus of 4-month-old *tbl/tbl* mice.** 1.5 μm thick sections show dark degenerative accumulations in the pyramidal cell somata (arrows in **B**). Condensed dark nuclei are occasionally observed within the pyramidal cell layer (arrowhead in **B**). Dark accumulations and vacuoles found throughout pyramidal cell cytoplasm have the same ultrastructural features (arrows in **C–E**) as those found in Purkinje cells and spinal motor neurons. Bars = 100 μm **(A)**, 20 μm **(B)**, 2 μm **(C)**, and 1 μm **(D–E)**.

**Figure 5:**
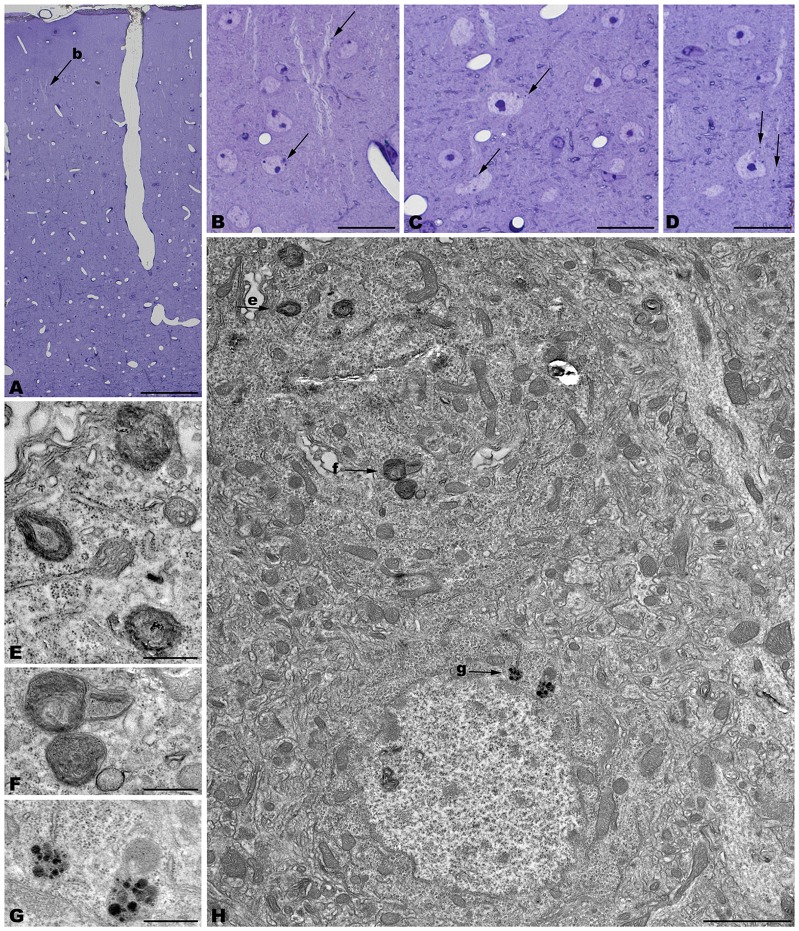
**Coronal sections through the frontal cerebral cortex of 4-month-old *tbl/tbl* mice.** 1.5 μm thick sections illustrate dark degenerative accumulations in both pyramidal cell somata (arrows in **B–D**) and apical dendrites (arrow in **B**). Cytoplasmic inclusion with characteristics of autophagosomes (arrowhead in **H**; **e**) and lysosomes (arrows in **H**; **f,g**) are observed within the pyramidal cell bodies. Bars = 100 μm **(A)**, 20 μm **(B–D)**, 2 μm **(E)**, and 0.5 μm **(E–G)**.

**Figure 6:**
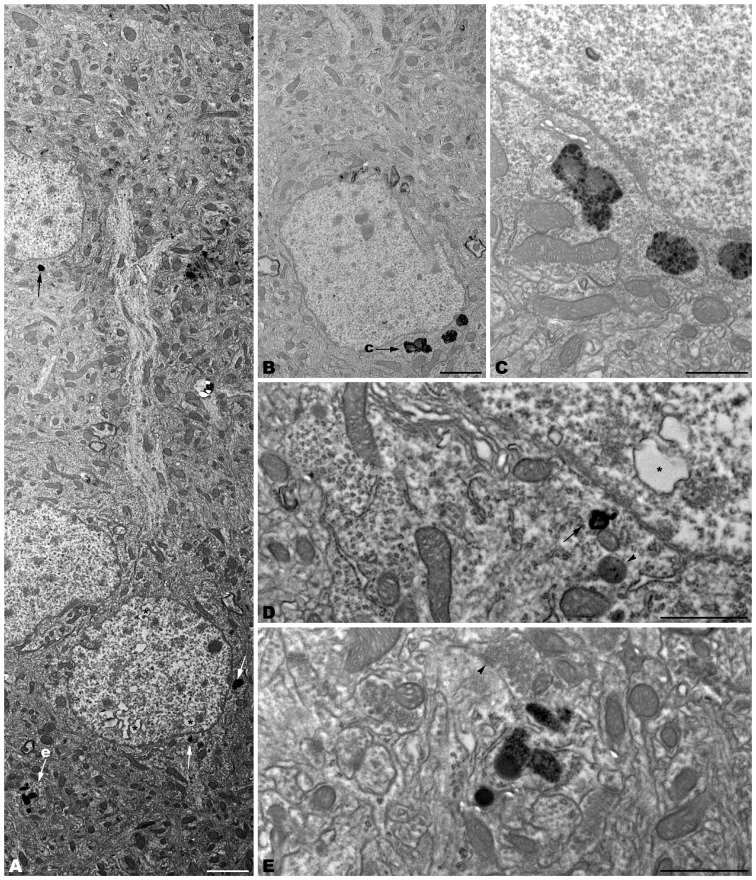
**Microphotographs of coronal sections through the frontal cerebral cortex of 4-month-old *tbl/tbl* mice.** Lysosomes located within the cytoplasm of pyramidal cell somata (arrows in **A**; **B–D**) and dendrites (arrow in **A**; **e**) which receive synapses of normal appearance (arrowhead in **E**). Some pyramidal cells with lysosomes in their cytoplasm show vacuoles within their nuclei (asterisks in **A,D**). Bars = 2 μm **(A,B)** and 1 μm **(C–E)**.

The electron microscopic study of *tbl/tbl* brain showed a similar morphological characteristics for the vacuoles accumulated in the cytoplasm of the neurons of the different brain areas analyzed. Features demonstrative of all the phases of the autophagic process—from early double-bounded autophagic vacuoles (autophagosomes) to dark final autolysosome (Clarke, 1990; Dunn, 1990a,b)—are observed within the cytoplasm of the cell bodies of Purkinje cells (Figures 1C–F), spinal cord motor neurons (Figure 3), CA3 pyramidal neurons (Figures 4C–E), and neocortical pyramidal neurons (Figures 5E–H, 6A–D), and within their dendritic trees (Figures 3A, 6A,E). In semithin sections these vacuoles are observed as dense dark points (Figures 1A,B, 2C–E,I, 4B, 5B–D). Other cellular organelles are consistently found, for example multivesicular bodies (Figure 3C) and healthy mitochondria intermingled with mitochondria showing different stages of degeneration such as the blurring and loss of their crests, and the progressive darkening of the mitochondrial matrices (Figures 1, 3, 4, 5, 6).

The cerebellar cortex of *tbl/tbl*, in addition to Purkinje cell degeneration, shows an evident shallowing of the cytoplasm of glial processes surrounding Purkinje cell somata and dendrites (Figure 1). In the molecular layer, dark masses of debris resembling final autolysosomes are observed within the glial cytoplasm (Figures 1D,E). Rather than the product of a degenerative glial process, these cell inclusions are probably the morphological evidence of the homeostatic cleansing role played by glial cells in eliminating cellular debris resulting from the degeneration of the Purkinje cell dendrites.

The spinal cord—as was reported previously for the *tbl/tbl* cerebellum (Dusart et al., 2006), and now for the other brain areas analyzed here (data not shown)—exemplifies the progressive evolution of the formation of autolysosomes throughout the lifespan of the animals. Thus, in the youngest *tbl/tbl* mice very few motor neurons show scarce dark inclusions in their cytoplasm (Figure 2H). The number increased with age, with inclusions being observed in almost all the motor neurons in 4-month-old mice (Figure 2).

Alterations of the cell nucleus have been correlated with autophagy (Clarke, 1990; Dusart et al., 2006). Our study found—in addition to chromatin alterations in Purkinje cell nuclei (Dusart et al., 2006)—only two different nuclear alterations: (i) in the CA3 of the hippocampus, dark condensed nuclei throughout the pyramidal layer were occasionally observed (Figure 4B); and (ii) some pyramidal neocortical neurons, despite possessing an apparently normal chromatin, showed several isolated single-membrane empty vacuoles mainly located at the nuclear periphery (Figures 6A,D). However, no signs of a dramatic neuronal cell loss were observed in *tbl/tbl* spinal cord, CA3 area, or neocortex.

The neuropil of the anterior horn, CA3, and neocortex did not show obvious structural alterations, and the glial sheets surrounding neuronal bodies and prolongations—in contrast to the case of the cerebellar cortex (Figure 1) —had a normal appearance (Figures 3, 4, 5, 6). Similarly, axosomatic and axodendritic synapses established with the autophagosome/autolysosome-filled neurons observed here did not show degenerative changes (Figure 3).

**Figure 7:**
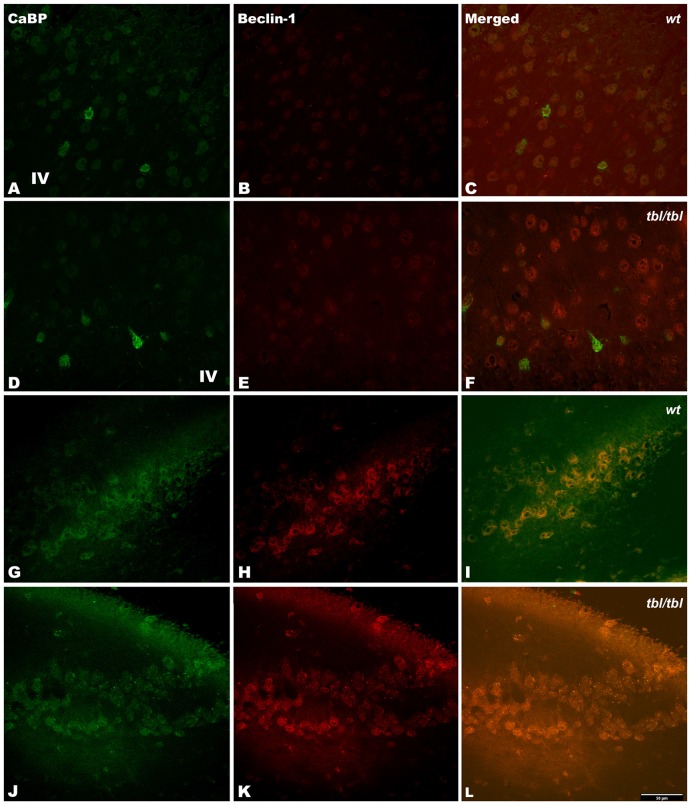
**Confocal images of coronal sections through the frontal cortex layers II-III (A–C) and CA3 hippocampus (G–I) of 4-month-old *wt* mice, and the same neocortical (D–F), and hippocampal region (J–L) of 4-month-old *tbl/tbl* mice.** Beclin-1 expression is most pronounced in *tbl/tbl* brains **(B,K)**, and as in *wt* co-express with calbindin (CaBP) inmmunoreactive and non-immunoreactive cell bodies **(C,F,I,L)**. IV, layer IV. Bars = 50 μm.

**Figure 8:**
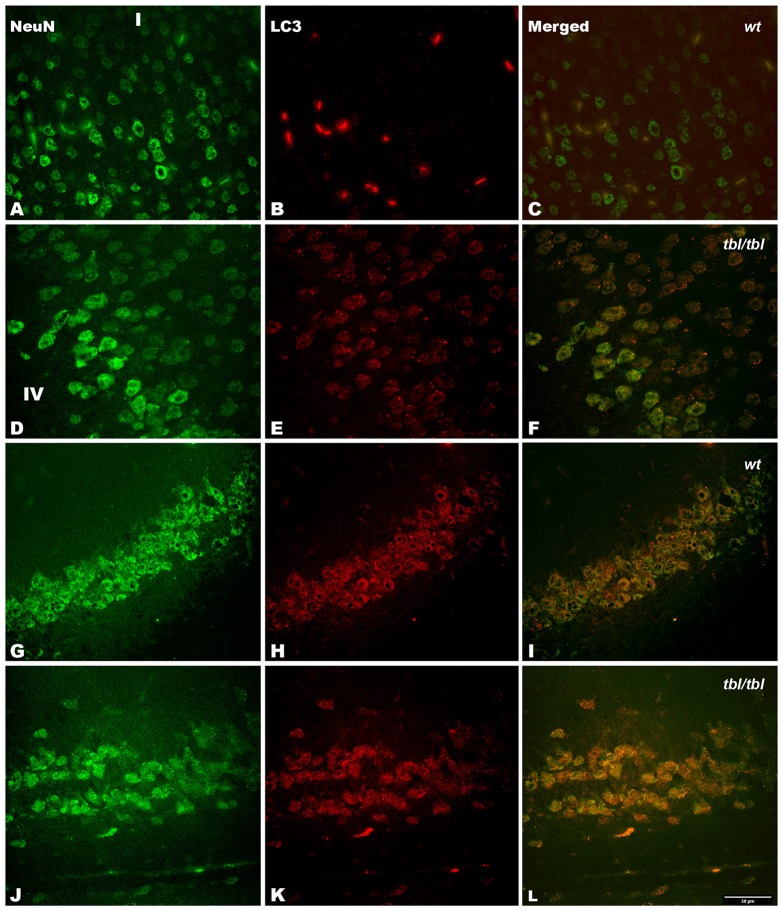
**Confocal images of coronal sections through the frontal cortex layers II-III (A–C) and CA3 hippocampus (G–I) of 4-month-old *wt* mice, and the same neocortical (D–F), and hippocampal region (J–L) of 4-month-old *tbl/tbl* mice.** Light chain 3 (LC3) co-expression with the neuronal marker NeuN is also most pronounced in *tbl/tbl* brains **(F,L)** than in *wt* similar brain regions **(C,I)**. I, layer I; IV, layer IV. Bar = 50 μm.

**Figure 9:**
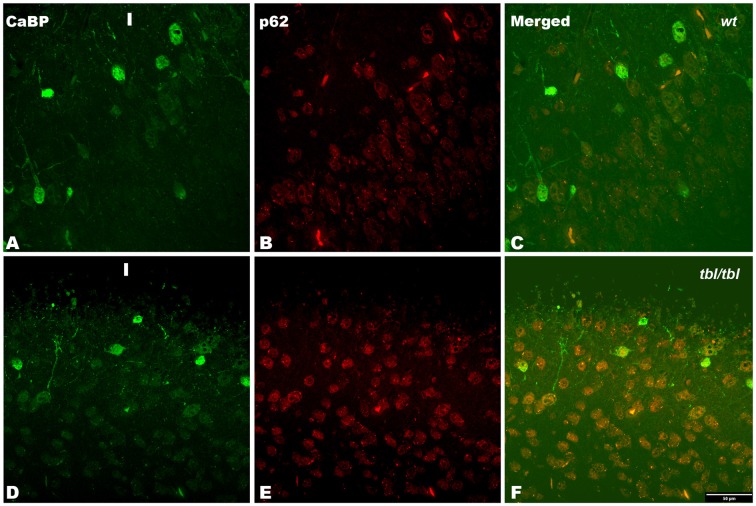
**Confocal images of coronal sections through the frontal cortex layers II-III (A–C) of 4-month-old *wt* mice, and the frontal cortex (D–F) of 4-month-old *tbl/tbl* mice.** p62 immunoreactive is stronger in *tbl/tbl* cortex **(E)** than in *wt* cortex **(B)**; and in both *wt* **(C)** and *tbl/tbl* **(F)** co-express in calbindin (CaBP) immunoreactive neuronal cell bodies. I, layer I. Bars = 50 μm.

The immunohistochemical study showed a prevalence of autophagy markers immunostaining in areas occupied by pyramidal cells as the pyramidal layer of the CA3 of the hippocampus and the layers II-III and V of the frontal neocortex (Figures 7, 8, 9), while layers I, IV and VI possessed a weak or occasionally inexistent labeling. The marker of the early stage of vesicle nucleation leading to the formation of autophagosomes beclin-1 (Kihara et al., 2001) was expressed in calbindin immunoreactive and calbindin non immunoreactive cell somata, and in all cases was more abundant in the cerebral cortex (Figures 7D–F) and the hippocampal CA3 region of *tbl/tbl* mice (Figures 7J–L) than in the same brain areas of *wt* mice (Figures 7A–C,G–I). The same immunohistochemical results were obtained by detecting the expression of the microtubule-associated protein—LC3, in which LC3 co-expressed with NeuN labeled somata more strongly in the cerebral cortex (Figures 8D–F) and hippocampal CA3 area (Figures 8J–L) of *tbl/tbl* mice than of *wt* mice (Figures 8A–C,G–I, respectively). Qualitatively, the most evident difference of the presence of autophagic vacuoles between *tbl/tbl* (Figures 9D–F) and *wt* (Figures 9A–C) mice was found in the detection of the expression of the LC3-phospatidylethanolamine complex binder p62 (Franchi et al., 2012), which is co-expressed in calbindin immunoreactive neuronal cell bodies and dendrites. Therefore, present immunohistochemical analyses of the autophagic vacuole formation cycle demonstrated the presence of a fine grained labeling within the neuronal cytoplasms (Figures 7, 8, 9) congruent with electron microscopy findings, which in all cases was more evident in the *tbl/tbl* than in *wt* brains.

**Figure 10:**
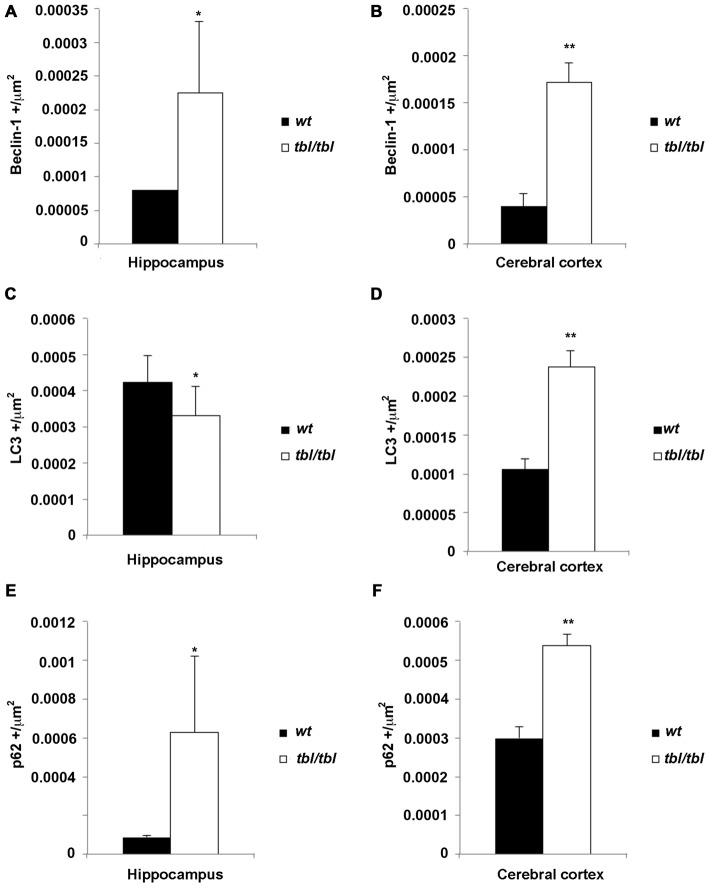
**Histograms of Beclin-1 (A,B), LC3 (C,D), and p62 (E,F) immunoreactivity densities through the CA3 hippocampus and frontal neocortex of 4-month-old *wt* mice (black bars), and the same brain regions of 4-month-old *tbl/tbl* mice (white bars).** In the hippocampus the differences of immunoreactivity densities are not statistically significant (asterisks), being *p* > 0.5 for Beclin-1, *p* = 0.440 for LC3, and *p* = 0.397 for p62. In contrast, the differences of immunorectivity densities are statistically significant for the three autophagic cycle markers in the cerebral cortex (double asterisks; *p* = 0.019 for Beclin-1, *p* = 0.019 for LC3, and *p* = 0.017 for p62).

The density of the immunoreactivity for the three markers of the autophagic cycle studied here showed different results according to the analyzed area. Thus, in the CA3 of the hippocampus although the densities of beclin-1 and p62 immunoreactivity were higher in *tbl/tbl* mice than in *wt* ones, their values were under the level of significance (Figures 10A,C,E). On the contrary, *p* values showed statistic significances in the frontal cortex for the immunoreactivity densities of the three markers, which were higher in *tbl/tbl* than in *wt* mice (Figures 10B,D,F). Although most detailed analyses by using western blot of each specific brain area are needed, present data reinforces the qualitative differences of the autophagic cell cycle observed in the mutation *tambaleante*.

## Discussion

Autophagy is a cellular physiological mechanism that maintains cellular—and hence, neuronal—homeostasis by delivering damaged organelles and cytosolic metabolic products to the lysosomes for their degradation (for a review, see Lim and Yue, 2015; Nikoletopoulou et al., 2015). During development, autophagy plays a role in the programmed cell death process which refines the definitive number of neurons (Bredesen et al., 2006; Wu et al., 2015). In addition to its homeostatic role; a protective role of baseline autophagy (Mizushima, 2005) has been implicated in processes such as the astroglial response to inflammation, the dendritic growth, the maintenance of adult neural stem cells, and synaptic plasticity (Kowalski et al., 2013; Nikoletopoulou et al., 2015). The deregulation of autophagy has been implicated in the pathogenesis of a number of neurodevelopmental diseases and adult neurodegenerative disorders (for a review, see Ghavami et al., 2014; Nikoletopoulou et al., 2015; Wu et al., 2015). Another essential mechanism for neuronal homeostasis is the protein degradation through the UPS pathway, whose alteration has also been implicated in the pathogenesis of several neurodegenerative diseases (de Vrij et al., 2004; Upadhya and Hegde, 2005; Rubinsztein, 2006; Hegde and Upadhya, 2007; Rusmini et al., 2007; Ramser et al., 2008; Chen et al., 2009; Deng et al., 2011; van Tijn et al., 2012; Dlamini et al., 2013). The two processes are linked in their collaboration in cell proteostasis; in fact autophagic receptors interact with UPS via the ubiquitin association domain (see Lim and Yue, 2015), and therefore the increase in autophagic activity observed here could be explained as the final expression of a deregulation of the UPS as occurs in *tbl/tbl* mutation (Mashimo et al., 2009).

Autophagy can be detected by different methods (Uchiyama et al., 2009; Li et al., 2014). Of these, the ultrastructural criteria defined by Clarke (1990) have to date proven to be one of the most useful. Thus, the observation of double-membrane-bound autophagic vacuoles—isolated or fused with lysosomes—is a clear indication of an autophagy active at the moment of fixation (see Figures 1, 3 in Clarke, 1990). This evidence is ratified by our immunohistochemical analyses, in which the cytoplasmic location of markers of the autophagosomes cycle (see Franchi et al., 2012) coincides with those of the autophagic signs detected under the electron microscope. Earlier analysis dealing with the different mechanisms of cell death involved in mutant mice losing Purkinje cells demonstrated that *tbl/tbl* Purkinje cells died by autophagy (Dusart et al., 2006). Our results confirm and extend these observations to other neuronal cell types such as neocortical pyramidal neurons, CA3 hippocampal pyramidal neurons, and spinal cord motor neurons, and suggest that the damage of UPS elicited by HERC1 E3 ubiquitin ligase is not circumscribed only to the cerebellum, but affects other brain regions. Immunohistochemistry allows an overall plane of observation greater than electron microscopy regarding the type of neurons that are affected by the mutation. The few or none detection of autophagy markers in layers mostly occupied by interneurons such as the layer IV of the cerebral cortex (for the cerebellum, see Dusart et al., 2006), in contrast with its abundance in layers II-III and V of the cerebral cortex in which pyramidal neurons are numerous, and the pyramidal layer of the CA3 of the hippocampus; together to the co-expression of analyzed markers with NeuN expressing cell bodies, open the possibility that one of the main targets of the mutation would be projection neurons. In fact, the ultrastructural features of autophagy-affected neurons observed here—i.e., characteristics of their chromatin, disposition of the Golgi apparatus and the rough endoplasmic reticulum, the typical morphology of the emergence of their apical dendritic shafts, etc. (see Peters et al., 1991)—corresponds to projection neurons.

Evidence points to the fact that protein homeostasis is affected by both synaptic and neuronal activity (for a review, see Lim and Yue, 2015). In addition, differences in autophagy have been found between several types of neuron (Lim and Yue, 2015; Nikoletopoulou et al., 2015). Among the neurons most sensitive to induced autophagy are the cerebellar Purkinje cells (Dusart et al., 2006; Yue, 2007; Yue et al., 2008), the cortical neurons (Li et al., 2014), and the spinal cord motor neurons (Garcera et al., 2013; Zhang et al., 2013), the same neurons that are observed here. Taking these data together, it could be hypothesized that neurons receiving strong and varied inputs, and that possess a strong (intense) synaptic activity—as is the case of the projecting neurons observed here—will be more sensitive to *tbl/tbl* mutation than the rest of the neural cells. The vacuolization of the neuronal nucleus has been associated with several pathological conditions (see Sasaki et al., 2014), and may be related to the alteration of normal Ca^2+^ homeostasis (Mattson et al., 2000; Alonso and García-Sancho, 2011). Therefore, it is possible to argue that an alteration in the homeostatic role of the autophagy in neurons receiving strong glutamatergic inputs (i.e., cortical pyramidal neurons) could affect neuronal cytoplasmic-nuclear relationships, ultimately promoting cell degeneration.

One of the characteristics of *tbl/tbl* mutation is the dramatic loss of Purkinje cells (Wassef et al., 1987; Rossi et al., 1995; Dusart et al., 2006; Mashimo et al., 2009; Porras-García et al., 2013). Present results support previous observations and underline an important difference between the cerebellum and the other central nervous system regions analyzed. Thus, no clear signs of a significant neuronal cell loss were observed in the cerebral cortex, the hippocampus, or the spinal cord. Purkinje cells are among the neurons most commonly affected by spontaneous mutations (Sotelo, 1990; Dusart et al., 2006; for a review, see Porras-García et al., 2013). Furthermore, directed mutations to components of the autophagic pathways present motor symptoms and Purkinje cell degeneration (Komatsu et al., 2006; Liang et al., 2010). In fact, on the basis of the autophagic reactions found in cerebellar mutant mice, Yue et al. (2008) proposed that neurons could develop axonal local autophagic processes as regulatory mechanisms that, in neurodegenerative diseases, could finally induce neuronal death. Thus, in *Lurcher* mutants —in which Purkinje cells die as a result of the excitotoxicity elicited by the activity of mutated glutamate receptor Δ2 (GluR Δ2^Lc^; Zuo et al., 1997; Yue et al., 2002)—there is an abnormal accumulation of autophagosomes in Purkinje cell axons before cell degeneration (Wang et al., 2006). This result seems to be consistent with the almost complete absence of autophagosomes, despite the axonopathy and subsequent Purkinje cell degeneration found in autophagic *Agt7*-gene-deficient mice (Komatsu et al., 2007). A common characteristic of both mutant mice is the presence of axonal swellings in Purkinje cells (Wang et al., 2006; Komatsu et al., 2007; Yue, 2007; Yue et al., 2008). These axonal swellings—axonal torpedoes—were also found in *tbl/tbl* Purkinje cells before they died and disappeared (Rossi et al., 1995; Porras-García et al., 2013). Therefore, our earlier and present observations are in accord with the hypothesis of Yue et al. (2008), and would indicate that, at least in Purkinje cell autophagic degeneration, there is a process of axonal damage before neuronal cell death, irrespective of the nature of the mutation. Whether autophagic signs found in the rest of the projecting neurons analyzed here indicate that autophagy is playing its homeostatic role and ultimately slowing down or inhibiting neuronal death needs further analysis.

In conclusion, our results extend the effect of the HERC1 E3 ubiquitin ligase-*tbl* mutation to projecting neurons of the cerebral neocortex, the CA3 of the hippocampus, and the ventral horn of the spinal cord, which seem to be less sensitive than Purkinje cells to the mutation. Further behavioral and morphometric studies are needed to elucidate the degree to which their neuronal circuits are affected in *tbl/tbl* mutant mice.

## Author Contributions

RR and EMP-V contributed equally in this research. SB performed the confocal and statistical analyses. All authors had full access to all the data in the study and take responsibility for the integrity of the data. RR, EMP-V, and JAA conceived and designed the study. RR, EMP-V, and JAA performed the experiments. RR and EMP-V were the primary contributors to the electron microscopy observations. SB and JAA performed the confocal analyses. SB performed the statistical analysis. JLR performed and bred the tambaleante mutation. JAA prepared the figures and wrote the article. All authors approved the article.

## Funding

Authors are supported as follows: RR (Juan de la Cierva contract JCI-2011-08888 from the MINECO and VPPI-US from the University of Seville), EMP-V (DGICYT BFU2011-27207, and Spanish Junta de Andalucía CTS-2257), SB (Fundación Ramón Areces), JLR (Spanish Ministerio de Ciencia e Innovación Grant BFU2011-22498), and JAA (Subv. CEICE Excelencia 2012 BIO 1388, Delgado García, José María).

## Conflict of Interest Statement

The authors declare that the research was conducted in the absence of any commercial or financial relationships that could be construed as a potential conflict of interest.
